# 

**Published:** 1810-10

**Authors:** 


### Medical and Physical Journal.

VOL. XXIV.] OCTOBER, 1810. [NO. 140.

Printed for R. PHILLIPS, by E. Hemsted, Great New Street, Fetter Lane, London.

*THE Proprietor of the MEDICAL AND PHYSICAL JOURNAL respectfully announces, that he has made arrangements for securing to this work increased activity and interest. The value of the Communications must, as heretofore, depend on the liberality of the Faculty; and on the opinion entertained of the superiority of this method of disseminating facts and opinions, over the fugitive, expensive, and less efficacious one of printing tracts and pamphlets; and in the respect, it is simply necessary to state, that the circulation of this JOURNAL is universal wherever the English language is read. It is in future intended to devote a larger portion to foreign Medical Ltiterature, and to give more effect to the department of criticism. The article of intelligence will also receive an encreased interest.*

*The aid of all the correspondents and friends of this work is requested in cooperation of the intentions and designs of its Proprietor, and there will then be no doubt, but the MEDICAL JOURNAL will continue to serve as the acceptable Gazette of the Faculty in all parts of the world.*

*The name of a new Editor will appear in the next Number.*

### *ADVERTISEMENT.*

IN undertaking to conduct the Medical and Physical Journal, the present Editors think it incumbent on them to express their sincere desire to promote the objects of Medical Science. This Journal, from its commencement, has experienced a degree of patronage which assures them, that a publication which conveys the earliest information upon medical subjects, is well received, if it be ably and faithfully executed.—The advantage of obtaining monthly, in the remotest provinces, intelligence of every thing that is interesting to the profession, almost as soon as it transpires in the Capital, and of receiving accounts of medical affairs from every part of the civilized world, is too obvious to require demonstration: but on this account, allowance must be made, if the communications are not always of equal importance. The nature of the work scarcely admits of elaborate essays, or of profound and extended critiques. These must be sought for in the transactions of societies, in the annual, and in the quarterly medical publications, which are conducted in a manner, and with a spirit, that evince a decided advance in the art of late years in this country, where improvement in practice, and discoveries in science, are continually rewarding the exertions of the experimenter.

The views of the Editors of the Medical and Physical Journal will, therefore, be steadily directed to give publicity to such cases and facts as must interest every man who is desirous of being acquainted with what is passing in the medical world, and they trust that the value of these will augment, by the number of their correspondents becoming more respectable.

In the critical department also, they hope in future to give a satisfactory account of such new medical publications as may be interesting; and in executing this part of their labour, they feel anxious to merit the character of being correct and impartial.

As discussion is calculated to improve knowledge and establish principles, the pages of the Journal will be open to the examination of opinions; but the Editors wish it to be understood, that they are decidedly hostile to personal allusion and illiberal disputation.

In the present state of continental affairs, they cannot hope to obtain regular supplies of foreign journals; but they will neglect no opportunity of making their readers acquainted with the medical literature of other nations, and in this month's Journal they have the satisfaction to insert some interesting articles from the New York Medical Repository, a work which enjoys high and well-merited reputation in America. Finally, they have to acknowledge the politeness of numerous professional friends who have offered to assist them in the prosecution of a work, of which the success materially depends on the character of the communications it contains.

